# Human Intestinal Spirochetosis Incompatible With Dysplastic Adenomatous Epithelium

**DOI:** 10.7759/cureus.23140

**Published:** 2022-03-14

**Authors:** Cullen Lilley, Joseph Grech, Emily Martinbianco, Xiuxu Chen

**Affiliations:** 1 Department of Pathology, Loyola University Chicago Stritch School of Medicine, Maywood, USA; 2 Department of Pathology, Loyola University Medical Center, Maywood, USA

**Keywords:** tubular adenoma, brachyspira pilosicoli, muc1, brachyspira aalborgi, intestinal microbiota, sessile serrated lesion, sessile serrated adenoma, colonic adenoma, brachyspira, human intestinal spirochetosis

## Abstract

Human intestinal spirochetosis (HIS) refers to the colonization of spirochetal bacteria in the human intestinal tract. HIS caused by *Brachyspira *spp. has been recognized for decades, but their pathological and clinical significance is largely unclear. The coincidence of dysplasia in adenoma or adenocarcinoma and HIS is very rare, and whether spirochetes can colonize on dysplastic epithelium remains controversial. Here, we report a case that showed abrupt abolition of mucosal surface fringe formation on a tubular adenoma (TA) and increased cytoplasmic MUC1 expression in the dysplastic epithelial cells compared with adjacent nondysplastic colonocytes. The findings support the hypothesis that the epithelial colonization of spirochetes is significantly reduced by dysplasia likely due to loss of microvilli, and an increase of epithelial MUC1 expression might contribute to reduced spirochetal colonization in colonic mucosa.

## Introduction

Human intestinal spirochetosis (HIS) refers to the colonization of spirochetal bacteria in the human intestinal tract, particularly *Brachyspira* species in the colorectum [1-3]. Since the introduction of the name *Brachyspira aalborgi* (*brachy*, short and *spira*, helix in Greek; *aalborgi*, Danish town Aalborg where the biopsy was taken) by Hovind-Hougen et al. in 1982 [2], two main lineages of *Brachyspira* species, *B. aalborgi* (including *B. ibaraki*, *B. hominis*, and unclassified species) and *B. pilosicoli*, have so far been identified by phylogenetic analysis of bacterial 16S rRNA gene, among which *B. aalborgi* accounts for the majority of cases in humans [2,3].

The coincidence of HIS and colorectal dysplastic or neoplastic lesions have been reported, but whether spirochetes can colonize on dysplastic/neoplastic epithelium remains controversial. The first convincing morphological evidence that spirochetes were incompatible with adenoma was reported by Coyne et al., by demonstrating that spirochetes heavily colonized normal colonic epithelium but did not attach the neoplastic epithelium of adjacent tubular adenoma (TA) or tubulovillous adenoma (TVA) [4-6]. However, several other studies contrasted this finding by showing that spirochetes were present on the dysplastic epithelia of tubular and villous adenomas (VA) [7-9]. Other studies reported coincident diagnosis of HIS and adenomatous polyps or carcinomas without specifying whether the spirochetes were present on the surface of dysplastic epithelia or were only seen on the surface of nondysplastic colonocytes surrounding the adenomas or adenocarcinomas [3,10]. These conflicting observations imply a more complex interaction between epithelial cells and spirochetes. The heterogeneity of the adenomas and adenocarcinomas, diverse strains of spirochetes, and other factors might variably contribute to this complexity.

A recent study showed that *Brachyspira* infection or related change in the intestinal microbiome may alter the mucin expression profile [11]. However, the underlying mechanism and possible alternative interpretation for this finding remain to be further analyzed. The rarity of the coincidence of HIS with adenomatous polyps and the existing discrepancy in the literature merit the current case report, which demonstrates direct morphological evidence of the incompatibility between spirochetal epithelial colonization and dysplastic epithelial cells.

## Case presentation

**Figure 1:**
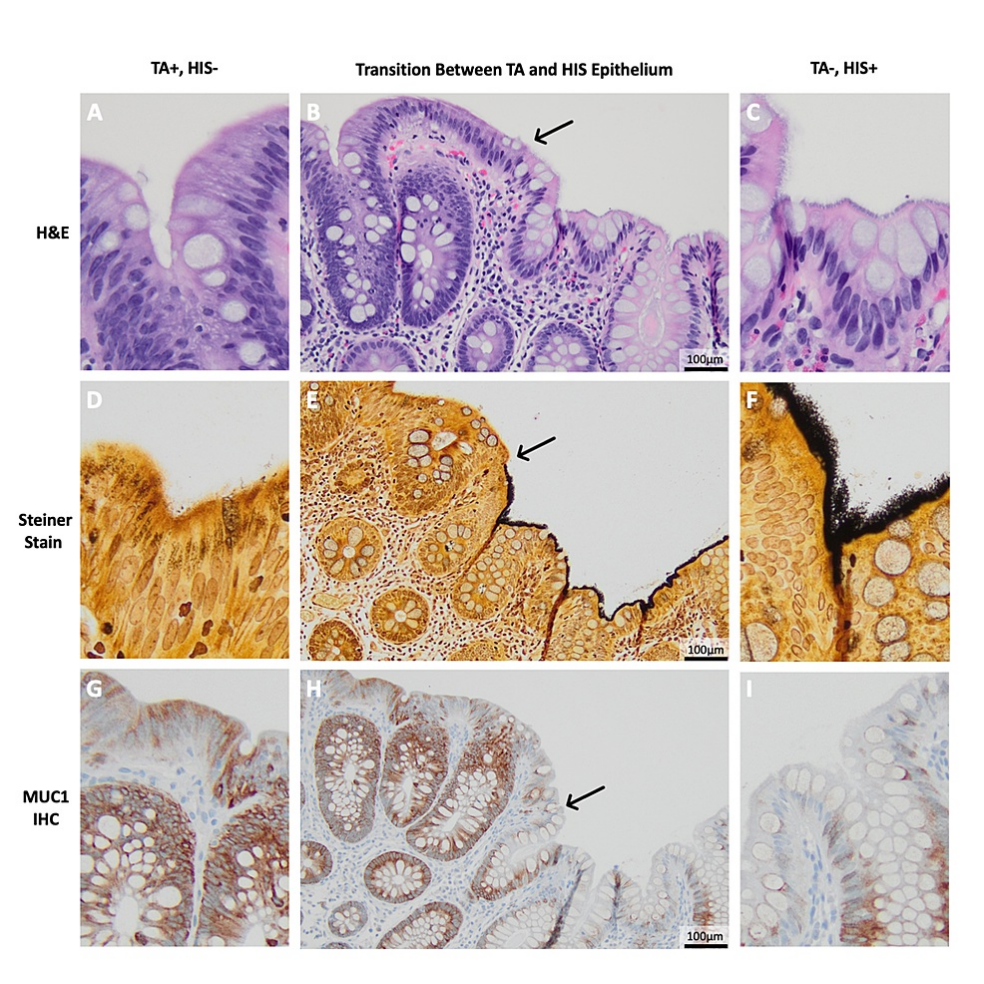
Spirochetal colonization at the interface between a tubular adenoma and non-dysplastic colonocytes Representative photomicrographs of the transitional zone (arrows) between dysplastic epithelium (TA) and adjacent non-dysplastic epithelium (normal colonocytes) (B, E, and H) by hematoxylin and eosin stain (A, B, and C), Steiner stain (D, E, and F), and immunohistochemical stain for MUC1 (G, H, and I). Representative higher power fields are shown for TA+, HIS- epithelium (A, D, and G) and TA-, HIS+ epithelium (C, F, and I). TA: tubular adenoma; HIS: human intestinal spirochetosis; IHC: immunohistochemical stain

A 48-year-old female with a past medical history of obesity, diabetes, hypertension, and HPV-positive cervical dysplasia presented to the gastroenterologist for an initial colonoscopy for cancer screening. She has a family history of colon and breast cancers. Prior to her colonoscopy, the patient was not experiencing any gastrointestinal symptoms, nausea, vomiting, diarrhea, hematochezia, hematemesis, melena, or abdominal pain. Her colonoscopy was significant for four polyps and several nonbleeding external hemorrhoids. Histologic examination of the removed polyps revealed two tubular adenomas and two hyperplastic polyps. One of the two tubular adenomas from the ascending colon revealed a Steiner stain-positive, basophilic "pseudo-brush border" on the non-dysplastic epithelia directly adjacent to the tubular adenoma indicative of HIS; however, the dysplastic epithelium of the tubular adenoma was unaffected by spirochetes with no fringe or "pseudo-brush border" formation. Endoscopic findings reported that this polyp was pedunculated with an irregular surface and a mottled green-tinged background epithelium. Immunohistochemical stain (IHC) for MUC1 revealed increased cytoplasmic MUC1 expression by the dysplastic epithelia in comparison with the background non-dysplastic colonocytes (Figure 1). Clinically, the patient preferred no antibiotic treatment because she remained asymptomatic, but she was recommended to follow up with any future symptoms or with regular surveillance if she remains asymptomatic.

## Discussion

Spirochetal microorganisms in the human intestinal tract have been studied for more than one century since an early report by pediatrician Theodor Escherich in 1884 [2,12]. However, it took decades for investigators to taxonomically distinguish the *Brachyspira *spp. (mainly *B. aalborgi* and *B. pilosicoli*) from other known pathogenic spirochetes, such as *Treponema* spp., *Borrelia *spp., and *Leptospira* spp. [1,2,4,13-16]. Until today, many important questions still remain to be answered, e.g., incidence and prevalence rates in the general population, transmission, pathogenesis, host-microbe interaction, pathological and clinical significance, standardized treatment algorithm, and optimal follow-up protocol for symptomatic patients. Some believe that these spirochetes are simply commensal microorganisms as seen in apparently healthy asymptomatic individuals, while others consider them as opportunistic or definitive pathogens responsible for acute and chronic gastrointestinal symptoms through zoonotic, foodborne, and waterborne transmission with increased risk in homosexual and HIV-positive men [3].

When first described by Lee et al. in 1971 [4] followed by Coyne et al. in 1995 [5], it was hypothesized that there was an unknown cellular or molecular mechanism that prevented *Brachyspira* spp. from colonizing the dysplastic adenomatous epithelium, since both studies showed sharp demarcation of spirochetal colonization at the interface between normal and dysplastic epithelia. However, even with the particularly unique demarcation of colonized mucosa terminating at the start of the dysplasia, there have been cases of spirochetal colonization of adenomatous polyps [7,8]. Calderaro et al. reported a case that was retrospectively found to have persistent HIS infestation eight years after surgery for colon adenocarcinoma. What is interesting is that spirochetes were identified on both non-dysplastic colonocytes and dysplastic epithelium of adenomas, but not on the transformed epithelial cells of adenocarcinoma [9]. A recent study reported that spirochetes were present in 80% (8/10) sessile serrated lesion/polyp (SSL/SSP) cases in a Japanese population, proposing a possible pathogenic association between HIS and SSL/SSP [17]. However, this association could not be confirmed in an Australian population and was believed to be most likely due to misdiagnosis of HIS [18]. Several other studies did not specify whether the spirochetes were present on the luminal surface of the adenomatous epithelium or not [3,10].

Most literature shows, as is consistent with our case, that the fringe formation or luminal surface epithelial colonization of spirochetes is significantly lower on dysplastic epithelial cells (TA, TVA, VA, or adenocarcinoma) than that on nondysplastic colonocytes (normal mucosa or hyperplastic polyp), with the exception of SSL/SSP, which appears more similar to the latter group.

One of the possible explanations for the reduced epithelial colonization by spirochetes was the loss of microvilli on the epithelial surface [5,9,19]. It was hypothesized that intact microvilli on epithelial cells were essential for spirochetal colonization, and loss or damaged microvilli would compromise the end-on attachment of spirochetes on epithelial cells. This hypothesis is supported by the early electron microscopic data [1,2,4-6,15]. However, one caveat for this hypothesis is that it did not include possible differences among various species of spirochetes. Although similar in aspects such as being slowly growing anaerobes and having slender tapered points, there are significant microbiological differences between the two main lineages of spirochetes, *B. aalborgi* and *B. pilosicoli*. *Brachyspira aalborgi* is one of the smallest *Brachyspira*, measuring 2-6 µm in length and 0.2 µm in diameter, with an estimated growth time of up to 2 weeks, and potentially pathogenic in humans, while *B. pilosicoli* (formerly *Serpulina* *pilosicoli* or *Anguillina coli*) is weakly beta-hemolytic, 4-20 µm in length and 0.2-0.5 µm in diameter, with an incubation time of six days, and likely pathogenic in humans, dogs, pigs, and poultry [16]. The latter was reported more densely colonized than *B. aalborgi* [3]. By comparing the prevalence of these two lineages in different populations in Australia, Brooke et al. found that *B. aalborgi* was the more common species in healthy urban individuals, while *B. pilosicoli* was prevalent largely in rural Aboriginal people and migrants from less developed countries [20]. Therefore, it is possible that the difference among various species might also affect their ability to colonize under certain conditions. Unfortunately, species identification was not performed in early studies because these specific species had not been taxonomically established at the time. For this reason, it would be interesting to correlate electron microscopic data with species in the future.

**Figure 2:**
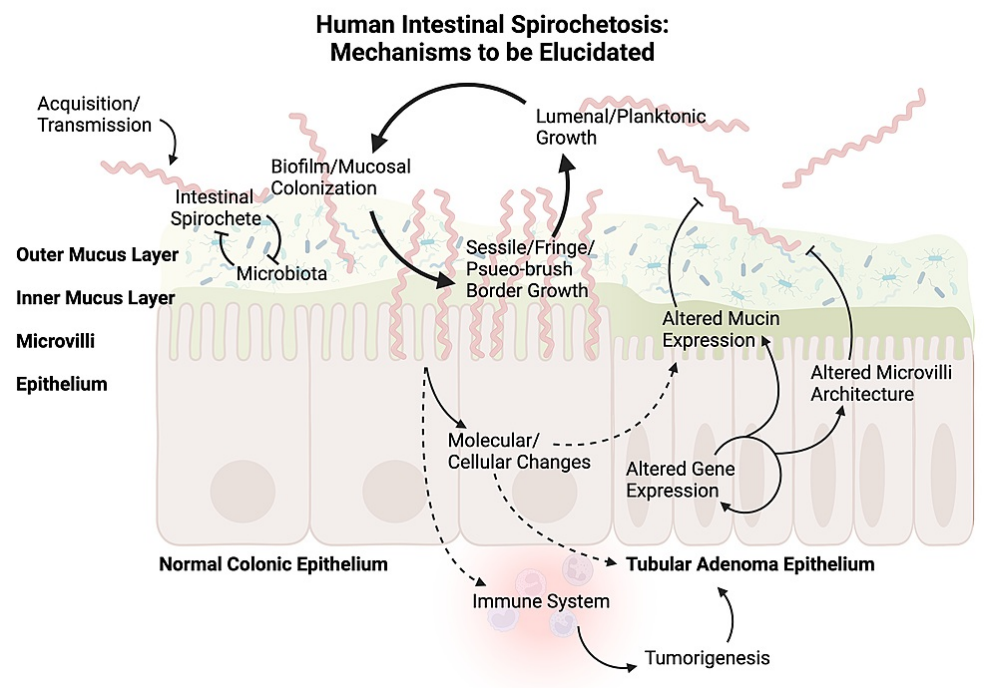
Schematic representation of possible mechanisms for epithelial colonization of human intestinal spirochetosis (HIS) The figure above illustrates the possible mechanisms of host colonocyte–spirochete interaction that have yet to be elucidated. The current understanding of this process is limited and is mainly based on scattered evidence from case reports and small series; therefore, many questions remain to be answered by both in vivo and in vitro studies. Dashed lines represent uncertain pathways with questionable directionality. Since current data cannot determine causal relationships between tumorigenesis and HIS, it is yet to be discovered if these bacteria have oncogenic properties or if their histologic comorbidity is only coincidental. Likewise, a possible causal relationship between mucin expression and spirochetal colonization also remains to be determined. (Figure created with *BioRender*.)

Adding to the complexity of this issue, in a case reported by Calderaro et al., spirochetes were absent on the initial adenocarcinoma but were identified on the adenomas and all normal colonic mucosa specimens during eight-year follow-up, highlighting that mucin expression is a dynamic process in the development of dysplasia and malignancy [9]. A recent paper from Ogata et al. observed an inverse relationship between the expression of MUC1 and HIS [11]. This is consistent with the finding in our case that upregulated MUC1 expression is associated with the absence of spirochetal fringe formation on adenomatous epithelial cells. However, the authors concluded that *Brachyspira* infection or a related change in the microbiome might have altered the mucin expression profile in humans. Considering the relative rarity of HIS in comparison with adenomas in humans and minimal inflammation in most HIS cases, a causal relationship in the above conclusion might not necessarily be true. Instead, changes of mucin expression, such as increased cytoplasmic MUC1 expression by the dysplastic epithelial cells in our case, may contribute to the reduced fringe formation by spirochetes (Figure 2). Further validation by future in vitro and in vivo studies is warranted.

## Conclusions

In summary, HIS caused by *Brachyspira* spp. has been recognized for decades, but their pathological and clinical significance is largely unclear. The coincidence of dysplasia in adenoma or adenocarcinoma and HIS is very rare. Our case showed abrupt abolition of mucosal surface fringe formation on a tubular adenoma and an increase of cytoplasmic MUC1 expression in the dysplastic epithelial cells in comparison with adjacent nondysplastic colonocytes. The findings support the hypothesis that the epithelial colonization of spirochetes is significantly reduced by dysplasia likely due to loss of microvilli, and an increase of epithelial MUC1 expression might contribute to reduced spirochetal colonization in colonic mucosa.
